# Twentieth Century Practice

**Published:** 1896-03

**Authors:** 


### Book Notices.

TWENTIETH CENTURY PRACTICE.—An International Encyclopedia of Modern Medical Science; by Leading Authorities of Europe and America. Edited by Thomas L. Stedman, M. D., New York City. In 20 volumes. Vol. VI, Diseases of the Respiratory Organs. New York: William Wood & Co. 1896.

This volume contains about seven hundred and fifty pages, and is devoted entirely to diseases of the respiratory organs. The opening chapter is by Dr. Prosser James, of London, on the subject of diseases of the nose. In the seventy-six pages covered by this subject, Dr. James discusses the various external and internal diseases of the nose, including, in the external diseases, acne, furuncle, verruca, hypertrophy, sebaceous tumors, næous, rodent ulcer, lupus, rhinoscleroma, epithelioma, etc., and in the internal diseases: acute, purulent, membraneous, chronic, hypertrophic and atrophic rhinitis, rhinorrhœa, epistaxis, polypi, fibromata, papillomata, enchondromata, osteomata, exostoses, malignant growths, abscess, hæmatomata, the various diseases of the septum, neuroses, foreign bodies, nasal diseases in general lesions, etc.

The second chapter is by Dr. Jonathan Wright, of Brooklyn, on diseases of the accessory sinuses of the nose. In this chapter the diseases of the antrum of Highmore, the frontal sinus, the ethmoidal sinus, and the sphenoidal sinus are discussed.

Diseases of the naso-pharynx and the pharynx, by Dr. E. J.

Moure, of Bordeaux, France, covers 104 pages, and is one of the best written and most instructive chapters in this volume. Dr. Moure also contributes an exhaustive article of some fifty pages on diseases of the tonsils.

Dr. Albert H. Buck, of New York City, has contributed the article on diseases of the ear, and his well known reputation, both as a teacher and writer on this specialty, is a sufficient guarantee of its superior excellence.

The editor was fortunate in securing Dr. Francke H. Bosworth to write the chapter on diseases of the larynx. Dr. Bosworth's article covers more than one hundred and sixty pages, and is written in his usual happy and instructive style.

The diseases of the trachea and bronchial tubes have received due consideration in an article of 137 pages by Drs. T. Grainger Stewart and George A. Gibson, of Edinburgh, Scotland.

Dr. Winslow Anderson, of San Francisco, deals with the subject of diseases of the lungs (excluding croupous pneumonia and tuberculosis). To the general practitioner this chapter is the most useful one in this volume, and Dr. Anderson has given the fullest care to its preparation. It is needless to say that his task was well done.

This entire volume gives evidence of the wisdom of the editor in the selection of his contributors, and of the most painstaking care and eminent fitness for this work, on the part of the learned medical men who have contributed the articles that go to make up the volume. H.

